# [Retracted] Suppressive effect of microRNA‑138 on the proliferation and invasion of osteosarcoma cells via targeting SIRT1

**DOI:** 10.3892/etm.2023.11794

**Published:** 2023-01-10

**Authors:** Zhenchao Yuan, Hao Mo, Ligen Mo, Juliang He, Zhenjie Wu, Xiang Lin

Exp Ther Med 13:3417–3423, 2017; DOI: 10.3892/etm.2017.4426

Following the publication of this paper, it was drawn to the Editors’ attention by a concerned reader that certain of the cell migration and invasion assay data shown in Figs. 2D and 4D, and the western blotting data shown in Fig. 3C were strikingly similar to data appearing in different form in other articles written by different authors who were based at different institutes. Owing to the fact that the contentious data in the above article were already under consideration for publication, or had already been published elsewhere, prior to its submission to *Experimental and Therapeutic Medicine,* the Editor has decided that this paper should be retracted from the Journal. The authors were asked for an explanation to account for these concerns, but the Editorial Office did not receive a reply. The Editor apologizes to the readership for any inconvenience caused.

